# NCOA5 induces sorafenib resistance in hepatocellular carcinoma by inhibiting ferroptosis

**DOI:** 10.1038/s41420-025-02473-1

**Published:** 2025-05-02

**Authors:** Shuang Gao, Lulu Fan, Huiyan Wang, Anqi Wang, Mengyao Hu, Lei Zhang, Guoping Sun

**Affiliations:** 1 Department of Medical Oncology, The First Affiliated Hospital of USTC, Division of Life Sciences and Medicine, University of Science and Technology of China, Hefei, Anhui 230001 China; 2 Department of Oncology, The First Affiliated Hospital of Anhui Medical University, Hefei, Anhui 230001 China; 3 Department of General Surgery, The Second Affiliated Hospital of Bengbu Medical University, Bengbu, Anhui 233080 China

**Keywords:** Oncogenes, Cell death, Cancer therapeutic resistance

## Abstract

NCOA5 has been identified as a crucial factor in the progression of hepatocellular carcinoma (HCC). This study investigates the expression of NCOA5 in HCC, revealing its significant overexpression in tumor tissues compared to healthy liver tissues, as evidenced by analysis of the TCGA dataset and RT-qPCR in patient samples. Higher NCOA5 levels correlate with poor overall survival, highlighting its role as a prognostic indicator. Furthermore, our findings suggest that elevated NCOA5 is associated with resistance to sorafenib, a common chemotherapeutic agent for HCC, as shown through analysis of publicly available datasets and the establishment of sorafenib-resistant HCC cell lines. Mechanistically, NCOA5 appears to inhibit ferroptosis in HCC cells by modulating glutathione peroxidase 4 (GPX4) levels. Knockdown of NCOA5 sensitizes resistant cell lines to sorafenib and induces ferroptosis by decreasing GPX4 expression. Additionally, NCOA5 regulation of GPX4 is mediated through the transcription factor MYC. In vivo studies further validate that targeting NCOA5 enhances the efficacy of sorafenib in resistant HCC models by promoting ferroptosis. Collectively, these findings underscore the potential of NCOA5 as a therapeutic target to overcome drug resistance in HCC, providing insights into its role in modulating treatment responses and patient prognosis.

## Introduction

Hepatocellular carcinoma (HCC) ranks as the third most deadly form of cancer globally [1]. There’s a recent rise in its incidence and fatalities in Western nations, in contrast to a downward trend in Asian regions [2–4]. The therapeutic landscape for HCC is multifaceted and continues to evolve as research advances our understanding of this complex disease [5, 6]. Treatment strategies for HCC are influenced by the stage of the cancer at diagnosis, the function of the liver, and the overall health of the patient. Advanced-stage HCC has traditionally been a challenge to treat due to the aggressive nature of the disease and the frequent presence of underlying liver dysfunction [7, 8]. Systemic therapies have become a focal point in this stage, particularly with the advent of targeted agents such as sorafenib, lenvatinib, and more recently, immunotherapies like nivolumab and pembrolizumab [9, 10]. These systemic treatments can offer improved survival and quality of life. Despite the progress in treatment options, HCC remains a challenging cancer to treat effectively, and the survival rates are not as high as with other cancers. The heterogeneity of the tumors, the underlying liver disease, and the development of resistance to therapies pose significant hurdles [11, 12]. As such, there is a substantial push for the development of personalized medicine approaches, to tailor treatments based on genetic, epigenetic, and proteomic characteristics of the tumor, with the aim of improving outcomes for patients with HCC.

Sorafenib stands as the pioneering FDA-approved tyrosine kinase inhibitor (TKI) designated for the treatment of advanced HCC [5, 13, 14], positioning it as the primary choice for patients battling this condition. It operates by inhibiting the activation of the mitogen-activated protein kinase (MAPK)/extracellular-signal-regulated kinase (ERK) pathway [15], thus hindering the proliferation of cancer cells through the suppression of Raf isoforms including Raf, Raf-1, and B-Raf [16]. Despite these mechanisms, sorafenib extends the overall survival (OS) of patients by only about 2.3 to 3 months, which falls short of anticipated clinical outcomes in individuals with HCC [13, 17]. The limited effectiveness of sorafenib in treating HCC is primarily attributed to the development of acquired drug resistance [13, 14, 18]. Nevertheless, it is believed that there are further mechanisms at play promoting resistance to sorafenib in HCC, indicating the complexity of tackling drug resistance in this context.

Nuclear receptor coactivators (NCOAs) are a family of transcriptional coregulators that interact with nuclear receptors to modulate gene expression, playing critical roles in physiological processes and disease progression, including cancer [19–21]. Among them, nuclear receptor coactivator 5 (NCOA5) has been known to mediate transcriptional regulation in cells through interacting with several nuclear receptors, including estrogen receptors and LXR [22]. The importance of NCOA5 in HCC development was first revealed experimentally in a genetically engineered mouse model [23, 24]. Recent research has increasingly focused on the observation that NCOA5 is overexpressed in various human cancers [25–27], extending beyond its normal physiological role in the reproductive system to affect a wide range of tissue types. Interestingly, NCOA5 exists in multiple variants, including a shorter isoform, SNCOA5, which shares an identical N-terminal sequence (~400 amino acids) with the full-length NCOA5 (579 amino acids). Importantly, SNCOA5 expression is significantly elevated in HCC tissues compared to adjacent healthy tissues, whereas non-shortened NCOA5 (non-SNCOA5) expression is reported to be reduced in approximately 40% of HCC cases [23]. This distinction is critical, as emerging evidence suggests that NCOA5 may act as a haplo-insufficient tumor suppressor, while SNCOA5 potentially promotes HCC progression. For instance, ectopic overexpression of NCOA5 has been shown to inhibit HCC cell proliferation and xenograft tumor growth [28], whereas studies involving CRISPR/Cas9-mediated knockout of NCOA5 in LM3 cells revealed inhibition of epithelial-to-mesenchymal transition (EMT). However, this knockout likely affected both NCOA5 and SNCOA5 simultaneously, confounding the interpretation of results [29].

Within the NCOA family, NCOA4 has recently garnered attention for its role in ferroptosis, a form of regulated cell death driven by iron-dependent lipid peroxidation [30, 31]. NCOA4 regulates ferritinophagy, a process that mediates the degradation of ferritin and promotes the release of iron, thereby linking ferroptosis to cellular iron metabolism [32, 33]. Given the critical role of ferroptosis in cancer therapy resistance and progression, exploring the interplay between NCOA4 and ferroptosis provides important insights into targeting iron metabolism for cancer treatment.

Taken together, the dual roles of NCOA5 and SNCOA5 in HCC, combined with the broader relevance of the NCOA family in cancer biology and ferroptosis, underscore the need for further studies to delineate the specific contributions of these isoforms. A clearer understanding of these mechanisms, particularly in the context of therapeutic resistance, may reveal novel strategies to improve HCC treatment outcomes.

Ferroptosis is a regulated type of cell death, triggered by iron-dependent reactive oxygen species (ROS) due to extensive lipid peroxidation [34, 35]. This process is controlled through several enzymatic reactions [36–38], which include the biosynthesis of phospholipids rich in polyunsaturated fatty acids (PUFA) such as phosphatidylcholine, phosphatidylethanolamine, and others, and the specific oxygenation of these lipids by lipoxygenases. The role of glutathione peroxidase 4 (GPX4) is crucial as it uses glutathione (GSH) to transform harmful lipid hydroperoxides into harmless lipid alcohols, thus preventing overoxidation and blocking ferroptosis [36, 39]. Beyond its role in preventing pathological cell death related to degenerative diseases and brain injuries, GPX4-mediated ferroptosis is vital for killing cancer cells in mammals [37, 40]. Blocking GPX4 to promote ferroptosis has become a potential therapeutic approach to induce death in cancer cells [37]. However, the details on how GPX4 stability is managed in cancer cells and its effects on tumor growth are still not fully understood.

In this work, we tried to depict the role of NCOA5 in regulating HCC drug resistance and progression. And the NCOA5-inhibited ferroptosis of HCC was investigated with their crosstalk being illustrated.

## Results

### NCOA5 plays important roles in HCC resistance and patient outcome

**Fig. 1:**
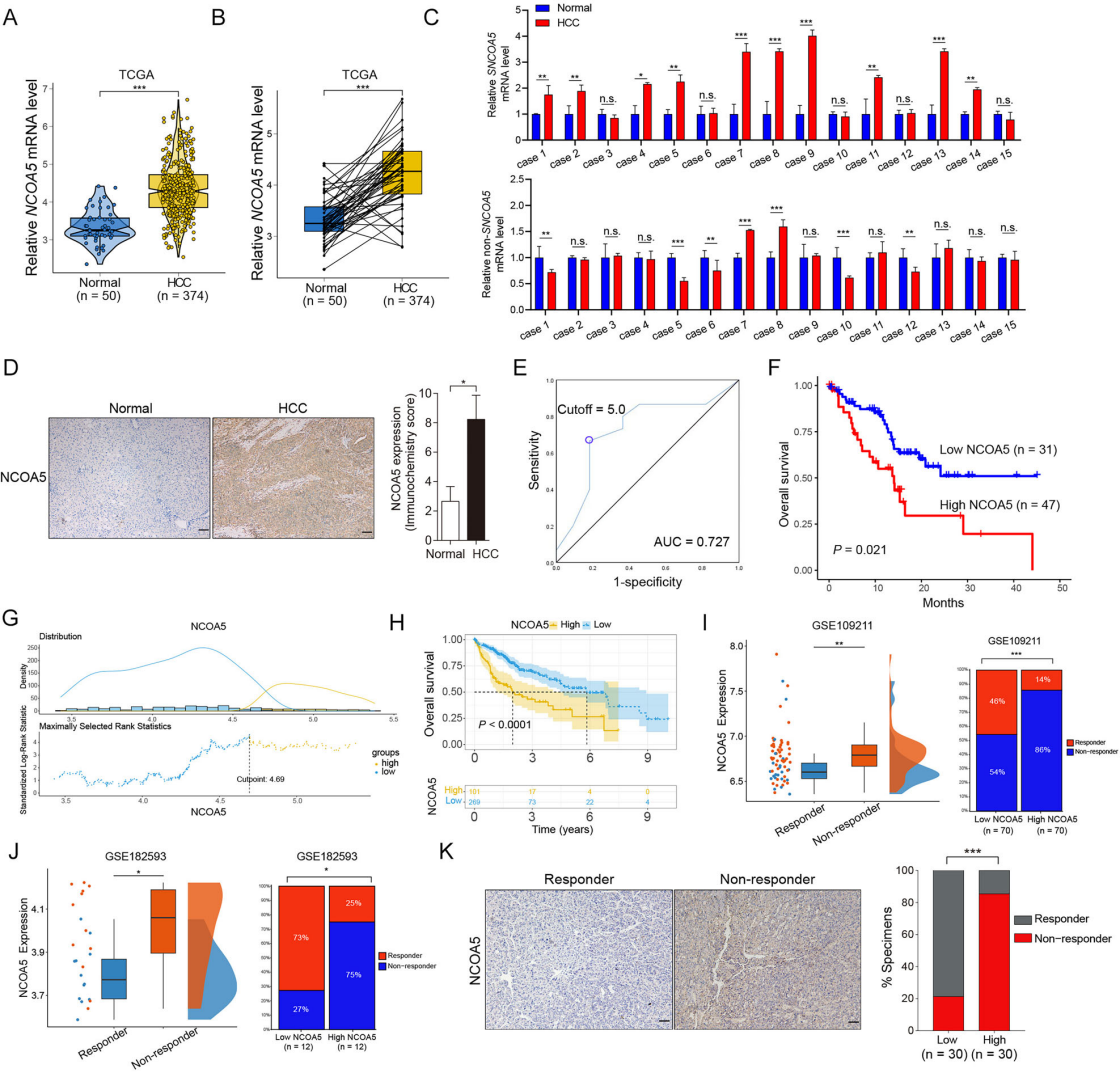
Elevated NCOA5 expression correlates with poor prognosis and sorafenib resistance in hepatocellular carcinoma (HCC). **A, B** Violin, box, and dot plots illustrating NCOA5 expression levels in the TCGA HCC dataset (TCGA-LIHC), comparing HCC tissues with peri-tumoral liver tissues. **C** RT-qPCR assays demonstrating mRNA levels of SNCOA5 and non-SNCOA5 in 15 HCC patient samples and their corresponding adjacent normal tissues. ***P < 0.001 by Student’s t-test. **D** Representative immunohistochemistry (IHC) images showing NCOA5 expression in HCC tissues versus paired adjacent normal tissues (left panel). Statistical analysis comparing IHC scores of 78 HCC tissues with their paired normal tissues (right panel). **E** Receiver operating characteristic (ROC) curve illustrating the overall survival (OS) predictive value of NCOA5 in 78 HCC patients. The area under the curve (AUC) is 0.727. The diagonal black line depicts a reference AUC of 0.5. **F** Kaplan-Meier analysis indicating the correlation between NCOA5 expression and OS in 78 HCC patients (*P* = 0.021 by log-rank test). **G** Distribution of risk scores and rank statistics generated from Kaplan-Meier analysis using the TCGA HCC dataset. **H** Kaplan-Meier analysis demonstrating significantly higher OS rates in the low-NCOA5 group compared to the high-NCOA5 group (*P* < 0.001 by log-rank test) using the TCGA HCC dataset. **I**, **J** Dot, box, and distribution plots showing NCOA5 expression in the publicly available GEO HCC datasets (left panels; **I**, GSE109211; **J**, GSE182593), comparing HCC patients who received sorafenib therapy, distinguishing between responders and non-responders. *P < 0.05, **P < 0.01 by Student’s t-test. Percentage of samples showing sorafenib responders or nonresponders relative to NCOA5 levels in HCC tissues (right panels; **I**, GSE109211; **J**, GSE182593). *P < 0.05 and ***P < 0.001 by χ² test. **K** Representative IHC images showcasing the differential expression of NCOA5 in HCC tissues of sorafenib responders versus non-responders (left panel). Statistical analysis showing the percentage of samples of sorafenib responders or non-responders relative to NCOA5 levels in HCC tissues (*n* = 60). ***P < 0.001 by *χ*^*2*^ test.

Previous research has indicated that NCOA5 is frequently overexpressed in hepatocellular carcinoma (HCC), yet its specific impact on the progression of this disease is not well understood. To explore the clinical relevance of NCOA5, we initially examined the expression levels of this gene using The Cancer Genome Atlas (TCGA) HCC dataset (TCGA-LIHC). Our analysis revealed that NCOA5 expression is notably higher in tumor tissues than in peri-tumoral liver tissues among a group of HCC patients (Fig. 1A, B). Considering the existence of an alternatively spliced form of NCOA5 mRNA, which encodes a shortened version known as SNCOA5, our TCGA analysis suggested that the combined expression of SNCOA5 and non-SNCOA5 is elevated in tumor tissues relative to healthy liver tissues in HCC patients. To validate these findings, we conducted RT-qPCR to estimate the mRNA levels of SNCOA5 and non-SNCOA5 in HCC patient samples. Among 15 HCC tissues analyzed, SNCOA5 mRNA levels were higher in 10 cases compared to their paired adjacent normal tissues, while non-SNCOA5 mRNA levels were lower in 5 cases (Fig. 1C). These results suggest that SNCOA5 plays a significant role in contributing to the overall increased expression of NCOA5 in HCC.

To determine the clinical relevance of NCOA5 in HCC, we conducted immunohistochemical analyses to compare the protein levels of NCOA5 (the antibody targeting both SNCOA5 and non-SNCOA5) in 78 HCC tissues with their paired normal adjacent tissues and found that NCOA5 is significantly higher in HCC tissues (*P* < 0.05; Fig. 1D; Supplementary Table 1). In addition, according to the receiver operating characteristic (ROC) curve (Fig. 1E) with an optimal cutoff point of 5.0 (H-score) for discriminating patients with worse overall survival (OS), we found that NCOA5 is overexpressed in 60.26% (47 of 78) of human HCC specimens. Furthermore, Kaplan-Meier analyses showed that patients with HCC with a higher level of NCOA5 are associated with worse OS (*P* = 0.021; Fig. 1F). In accordance, Kaplan-Meier analysis of TCGA database containing 360 HCC patients revealed that those with overexpressed NCOA5 have poorer prognosis (*P* < 0.001; Fig. 1G, H). These data strongly suggest that elevated levels of NCOA5 in HCC lead to a poor prognosis.

Chemotherapy resistance plays a key role in HCC progression. However, the role of NCOA5 in HCC drug resistance is not well understood. To explore the role of NCOA5 in regulating HCC drug resistance, we assessed the relationship between the levels of NCOA5 and sorafenib responsiveness. Across both two publicly available datasets from Gene Expression Omnibus (GEO), higher levels of *NCOA5* were significantly enriched in patients who failed to respond to sorafenib treatment (Fig. 1I, J). Consistently, by examining HCC tissue from our own cohort, in HCC patients with high expression of NCOA5, a significant enrichment for cases not responding to sorafenib was observed (Fig. 1K). These data altogether indicate that NCOA5 levels might be linked to sorafenib resistance in HCC.

### Aberrant NCOA5 expression promotes sorafenib resistance in HCC cells

**Fig. 2:**
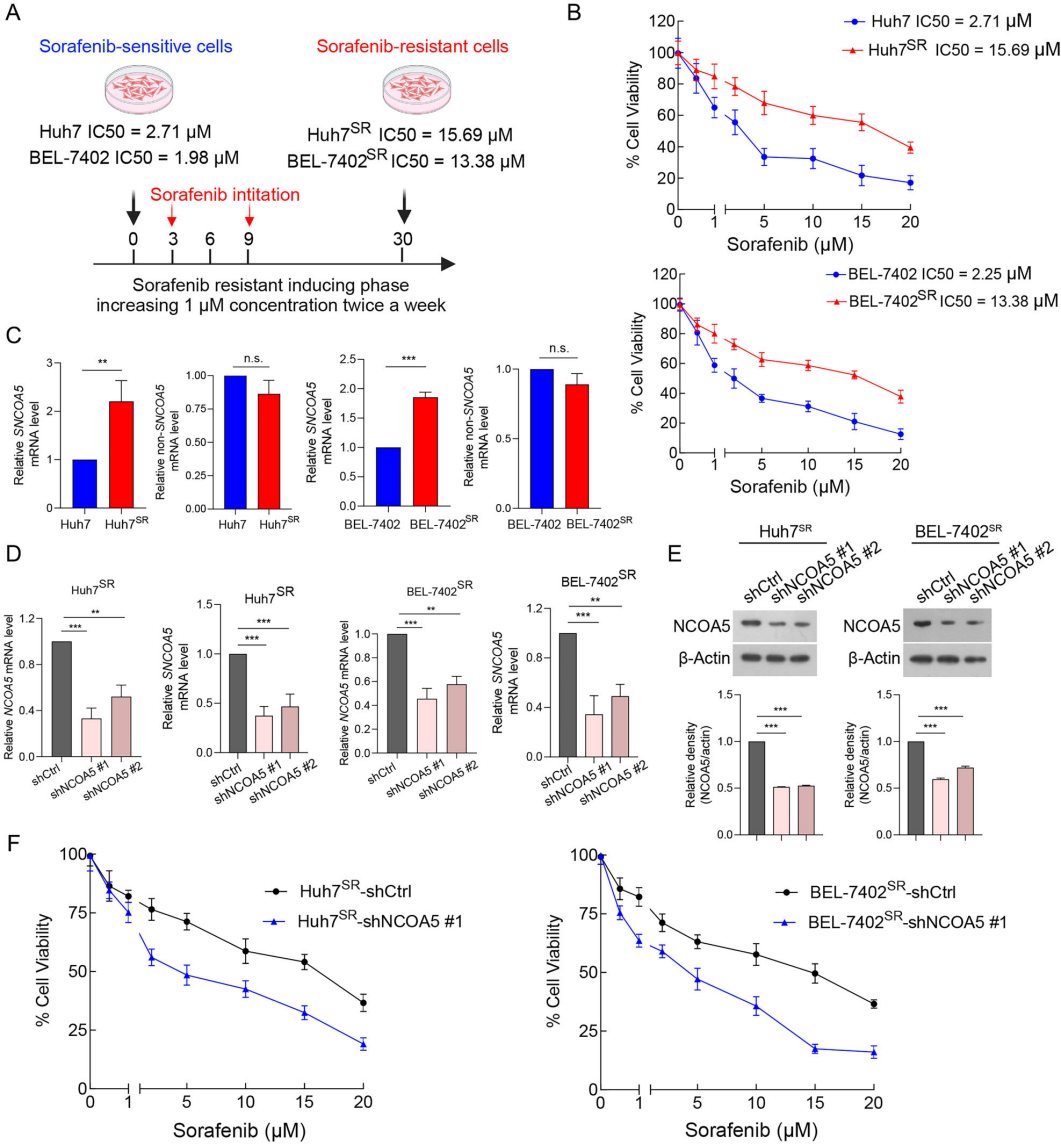
NCOA5 expression is increased in sorafenib-resistant HCC cells and contributes to resistance development. **A** Schematic representation of the establishment of sorafenib-resistant (SR) cells. **B** Cell viability assessed by CCK-8 assay following exposure to varying concentrations of sorafenib for 48 hours. Proliferation of both Huh7^SR^ and BEL-7402^SR^ cells was greater than that of their parental cells at different sorafenib concentrations. **C** Expression levels of SNCOA5 and non-SNCOA5 in Huh7^SR^ (left panel) and BEL-7402^SR^ (right panel) cells, as well as their parental cells, detected by RT-PCR and immunoblotting. **D** Expression levels of *NCOA5* ans *SNCOA5* in Huh7^SR^ (left panel) and BEL-7402^SR^ (right panel) cells transfected with shNCOA5 #1, shNCOA5 #2, or shCtrl, detected by RT-PCR. **P < 0.01, ***P < 0.001 by Student’s t-test. **E** Expression levels of NCOA5 in Huh7^SR^ (left panel) and BEL-7402^SR^ (right panel) cells transfected with shNCOA5 #1, shNCOA5 #2, or shCtrl, detected by immunoblotting. **F** Proliferation of both Huh7^SR^ and BEL-7402^SR^ cells transfected with shCtrl compared to those transfected with shNCOA5 #1 at varying concentrations of sorafenib.

To further establish the relationship between NCOA5 and therapy resistance in HCC, we generated Sorafenib-resistant HCC cell lines, called Huh7^SR^ (IC50 = 15.69 µM) and BEL-7402^SR^ (IC50 = 13.38 µM), as compared to their Sorafenib-sensitive parental cell lines Huh7 and BEL-7402 (IC50 of 2.71 and 1.98 µM, respectively) (Fig. 2A, B) [41, 42]. Interestingly, only SNCOA5 was elevated in sorafenib-resistant subclones of Huh7 and BEL-7402, Huh7^SR^ and BEL-7402^SR^ cells, whereas levels of non-SNCOA5 did not show differences in these cells (Fig. 2C). To determine whether NCOA5 plays a key role in sorafenib resistance, we conducted stable NCOA5 knockdown (shNCOA5, targeting both SNCOA5 and non-SNCOA5) cells in Huh7^SR^ and BEL-7402^SR^ (Fig. 2D, E). Remarkably, shNCOA5 sensitized these resistant cells to sorafenib treatment evidenced by cell viability, suggesting that SNCOA5 plays a critical role in mediating sorafenib resistance (Fig. 2F). These findings collectively demonstrate that NCOA5 suppression effectively counters sorafenib resistance in HCC.

### NCOA5 intercepts ferroptosis in HCC cells

**Fig. 3:**
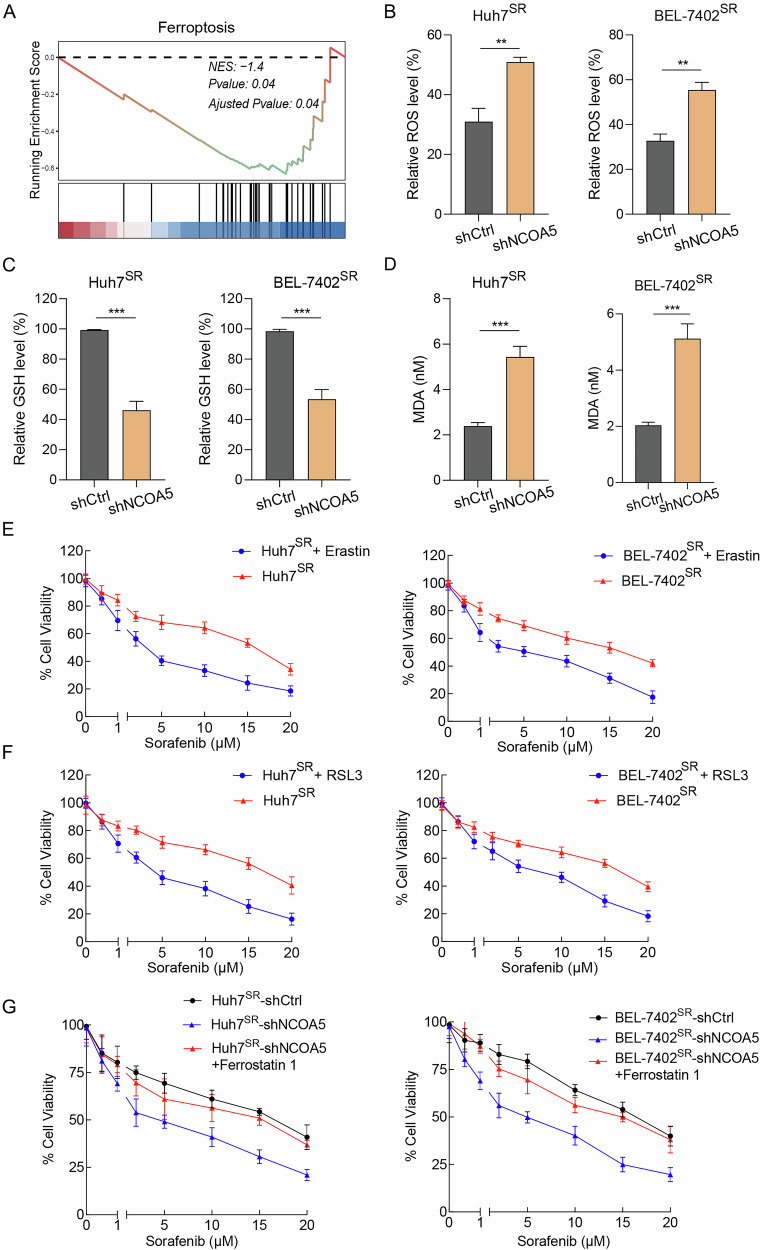
NCOA5 inhibits ferroptosis in HCC cells. **A** Gene Set Enrichment Analysis (GSEA) of ferroptosis-related genes, showing their enrichment in NCOA5 high versus NCOA5 low groups using the GSE182593 dataset. **B**–**D** Relative levels of reactive oxygen species (ROS) (**B**), glutathione (GSH) (**C**), and malondialdehyde (MDA) (**D**) in Huh7^SR^ (left panel) and BEL-7402^SR^ (right panel) cells transfected with shNCOA5 #1 compared to shCtrl. **P < 0.01, ***P < 0.001 by Student’s t-test. **E** Proliferation of Huh7^SR^ (left panel) and BEL-7402^SR^ (right panel) cells, with and without exposure to erastin. **F** Proliferation of Huh7^SR^ (left panel) and BEL-7402^SR^ (right panel) cells, with and without exposure to RSL3. **G** Proliferation of both Huh7^SR^ and BEL-7402^SR^ cells transfected with shCtrl compared to those transfected with shNCOA5 #1, with and without exposure to Ferrostatin 1.

To elucidate the potential mechanisms by which NCOA5 influences sorafenib resistance in HCC and HCC progression, we conducted Gene Set Enrichment Analysis (GSEA) on the dataset GSE182593 to explore the downstream signaling of NCOA5 and identified an inverse relationship between NCOA5 expression and ferroptosis signatures (*P* = 0.04, Fig. 3A). We further assessed reactive oxygen species (ROS) levels in Huh7^SR^ and BEL-7402^SR^ cells, revealing that shNCOA5 significantly increased intracellular ROS levels (*P* < 0.01, Fig. 3B). Additionally, we measured glutathione (GSH) and malondialdehyde (MDA) levels, key indicators of oxidative stress. GSH levels decreased (*P* < 0.001, Fig. 3C), while MDA levels increased (*P* < 0.001, Fig. 3D) in both Huh7SR and BEL-7402SR cells upon NCOA5 knockdown.

Next, we examined the effects of ferroptosis inducers and inhibitors to further confirm the role of NCOA5 in ferroptosis regulation. Treatment with ferroptosis inducers, erastin (10 μM) or RSL3 (1 μM), significantly reduced cell viability in NCOA5-knockdown cells compared to controls (Fig. 3E, F). Importantly, these effects were reversed upon treatment with the ferroptosis inhibitor ferrostatin-1 (1 μM) (Fig. 3G).

These results collectively demonstrate that NCOA5 attenuates intracellular ROS levels and oxidative stress, thereby inhibiting ferroptosis and contributing to sorafenib resistance in HCC. The observed modulation of ferroptosis by NCOA5 highlights its critical role in regulating cellular susceptibility to oxidative stress and ferroptosis-induced cell death.

### NCOA5 suppression induced ferroptosis via inhibiting GPX4 expression

**Fig. 4:**
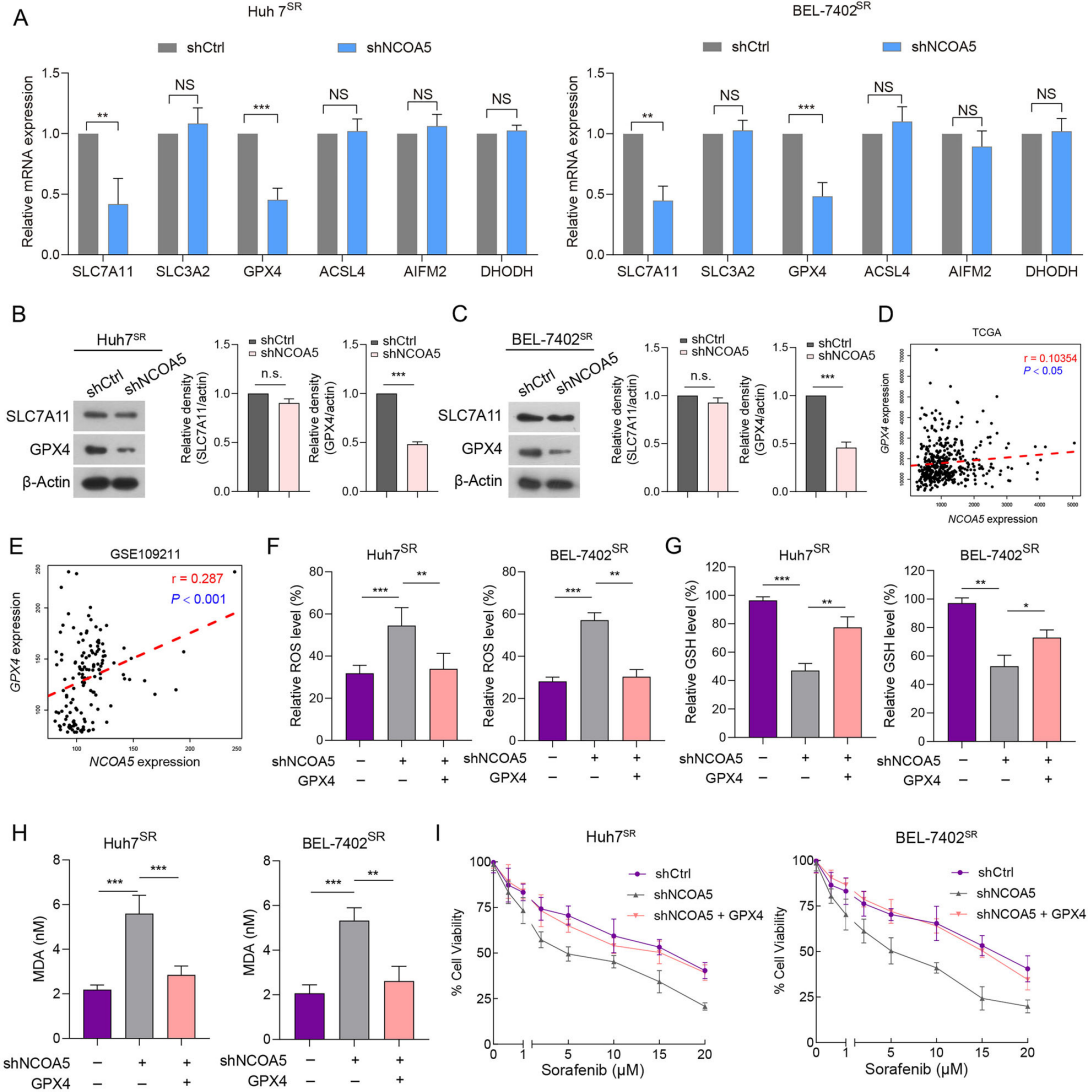
NCOA5 suppression induces ferroptosis by inhibiting GPX4 expression. **A** RT-qPCR assays demonstrated mRNA levels of indicated genes in in Huh7^SR^ (left panel) and BEL-7402^SR^ (right panel) cells transfected with shNCOA5 compared to shCtrl. NS, not significant; **P < 0.01, ***P < 0.001 by Student’s t-test. **B**, **C** Expression levels of SLC7A11 and GPX4 in Huh7^SR^ (**B**) and BEL-7402^SR^ (**C**) cells transfected with shNCOA5 compared to shCtrl, detected by immunoblotting. **D**, **E** Pearson correlation analysis of TCGA (**D**) and GEO (**E**) datasets revealed a significant positive correlation between NCOA5 and GPX4 expression. **F**–**H** Relative levels of ROS (**F**), GSH (**G**), and MDA (**H**) in Huh7^SR^ (left panel) and BEL-7402^SR^ (right panel) cells transfected with shNCOA5 #1 or GPX4 overexpression. *P < 0.05, **P < 0.01, ***P < 0.001 by Student’s t-test. **I** Proliferation of Huh7^SR^ (left panel) and BEL-7402^SR^ (right panel) cells transfected with shNCOA5 #1 or GPX4 overexpression.

To further elucidate the mechanism by which NCOA5 downregulation promotes ferroptosis, we examined ferroptosis-related proteins in Huh7^SR^ and BEL-7402^SR^ cells following stable NCOA5 knockdown. Quantitative RT-PCR assays revealed significant reductions in SLC7A11 and GPX4 mRNA levels, while other ferroptosis-related genes (SLC3A2, ACSL4, AIFM2 and DHODH) [43–45], remained unchanged (Fig. 4A). Western blot analysis further confirmed that NCOA5 knockdown resulted in a decrease in GPX4 protein levels, but did not affect SLC7A11 protein levels (Fig. 4B, C). These findings suggest that NCOA5 downregulation specifically targets GPX4 expression to promote ferroptosis in HCC cells. By examining The Cancer Genome Atlas (TCGA) and Gene Expression Omnibus (GEO) datasets, we revealed a significantly positive correlation between NCOA5 and GPX4 (Fig. 4D, E). Moreover, GPX4 overexpression inhibited the increased ROS levels induced by shNCOA5 (Fig. 4F) and reversed the effects on GSH and MDA levels (Fig. 4G-H). Notably, GPX4 overexpression abolished the sensitization of resistant cells to sorafenib treatment induced by NCOA5 knockdown (Fig. 4I). These findings suggest that NCOA5 reduction promotes ferroptosis by downregulating GPX4 expression.

### NCOA5 modulates the expression of GPX4 via the transcription factor MYC

**Fig. 5:**
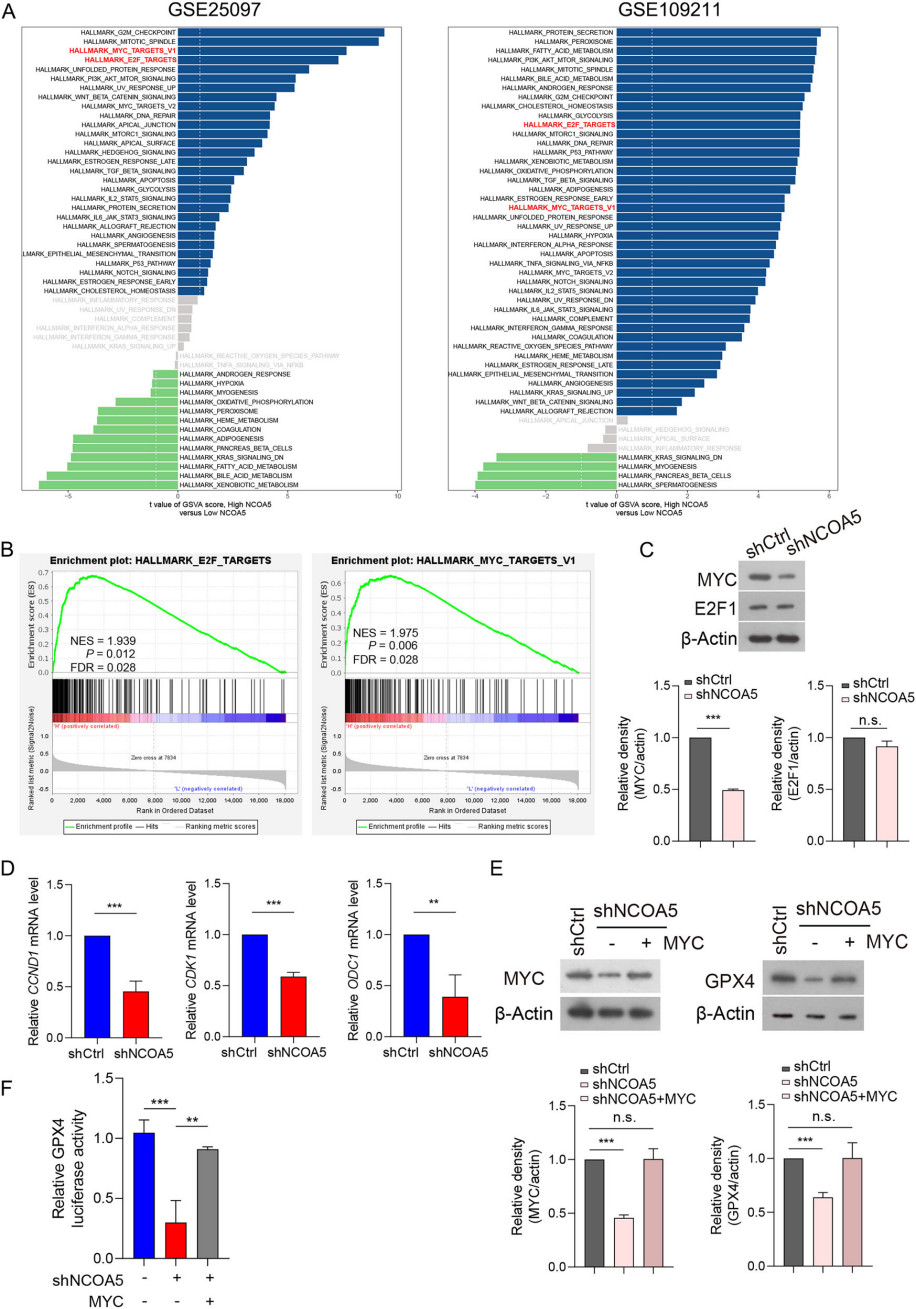
NCOA5 transcriptionally regulates the expression of GPX4 via MYC. **A** Differential distribution of signal pathway enrichment between the NCOA5 high versus NCOA5 low groups in GEO HCC datasets. **B** GSEA of E2F target genes (left panel) and MYC target genes (right panel), showing their enrichment in NCOA5 high versus NCOA5 low groups using the GSE25097 dataset. **C** Expression levels of E2F1 and MYC in Huh7^SR^ cells transfected with shNCOA5 compared to shCtrl, detected by immunoblotting. **D** Expression of these MYC targets in Huh7^SR^ cells transfected with shNCOA5 compared to shCtrl, detected by RT-qPCR. **E** Expression levels of MYC and GPX4 in Huh7^SR^ cells transfected with shNCOA5 (with or without MYC overexpression) compared to shCtrl, detected by immunoblotting. **F** GPX4 luciferase reporter activity in Huh7^SR^ cells transfected with shNCOA5 (with or without MYC overexpression) compared to shCtrl.

The regulatory mechanism of NCOA5 on GPX4 is not clearly understood. Given that NCOA5 has been reported to play a role in various signaling pathways across a broad range of cancer types, we decided to perform a gene set enrichment analysis (GSEA) on the published human hepatocellular carcinoma (HCC) expression profile (GSE1109211 and GSE25097). We found that high NCOA5 groups are enriched in these hallmarks, including G2M checkpoint signaling, MYC targets, E2F targets, mitotic spindle, PI3K AKT MTOR signaling, Estrogen Response, Glycolysis, DNA repair (Fig. 5A). We need to clarify how the expression of GPX4 is regulated by NCOA5. NCOA5 has been known to mediate transcriptional regulation in cancer. Therefore, the focus is on the signaling of transcription factors, such as MYC targets and E2F targets (Fig. 5B). MYC stimulates a variety of genes involved in cell growth, including E2F genes like E2F1 [46]. And several ferroptosis markers including SLC7A11, ATF4, GPX4, are regulated by E2F1 [47]. We then conducted western blot analysis confirmed that NCOA5 knockdown resulted in a decrease in MYC protein level, while did not affect E2F1 protein levels (Fig. 5C). To further confirm the role of MYC as a downstream mediator of NCOA5, we analyzed the expression of key MYC downstream target genes, including CCND1, CDK1, and ODC1, which are involved in cell cycle regulation and metabolism [48]. The results showed that the expression of these MYC targets was significantly reduced following NCOA5 knockdown, further validating the functional involvement of MYC (Fig. 5D). Further, the rescue experiment shows that overexpression of MYC can reverse the inhibitory effect of NCOA5 knockdown on GPX4 expression (Fig. 5E). To strengthen these findings, we investigated the effect of MYC overexpression in control (shCtrl) cells. The results showed that MYC overexpression alone significantly increased GPX4 protein levels (Fig. 5E). Additionally, luciferase reporter assays confirmed that GPX4 is a downstream target of MYC, as MYC mediated the decrease in GPX4 induced by NCOA5 knockdown (Fig. 5F). Mechanistically, our findings suggest that NCOA5 transcriptionally regulates MYC expression, and MYC, in turn, promotes GPX4 expression, thus inhibiting ferroptosis. These results establish a novel NCOA5-MYC-GPX4 signaling axis that underlies NCOA5-mediated ferroptosis resistance in HCC.

### NCOA5 reduction enhances sorafenib efficacy in treating resistant HCC in vivo

**Fig. 6:**
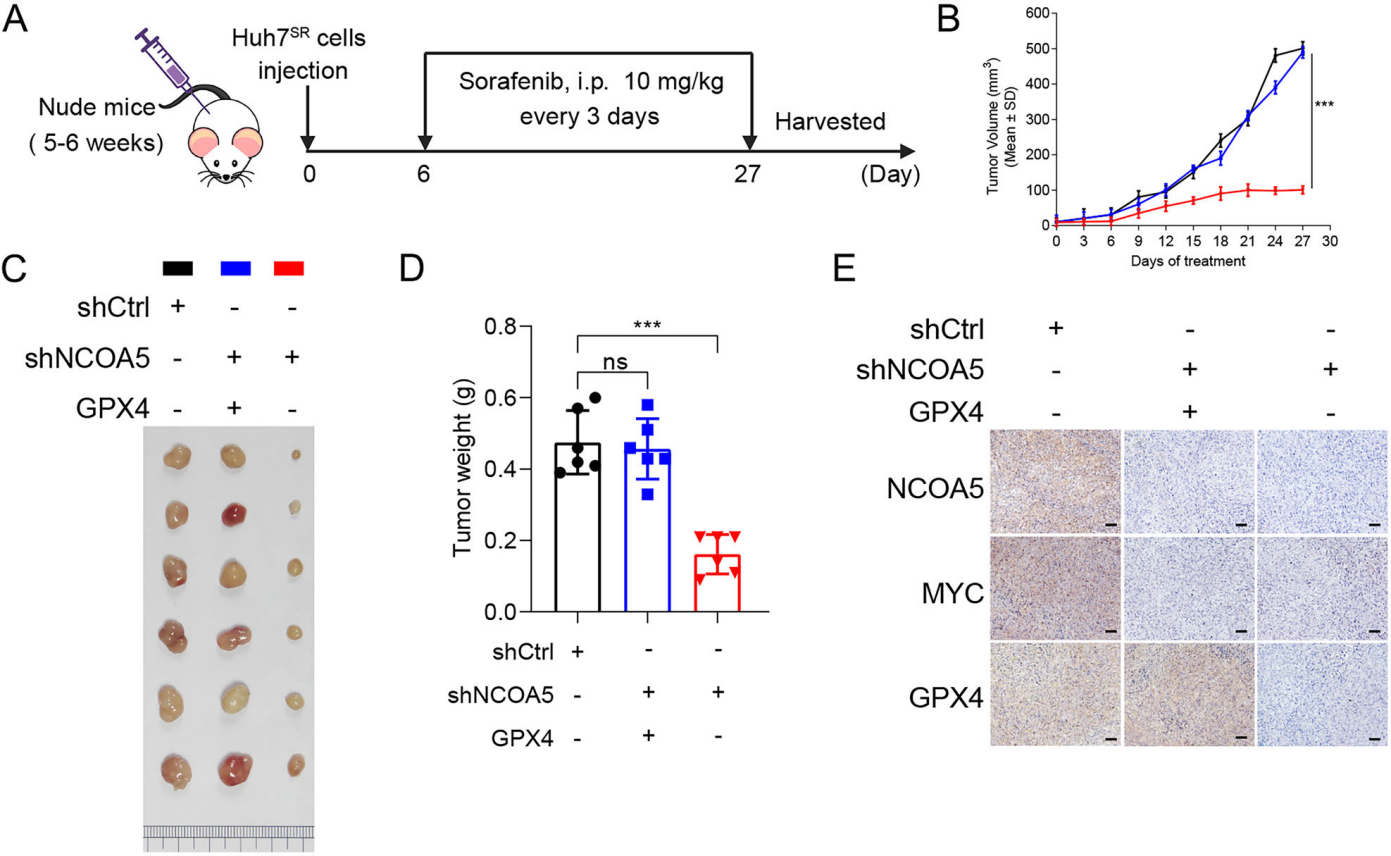
NCOA5 reduction enhances sorafenib efficacy in treating sorafenib resistant HCC in vivo. **A** Treatment schedule illustrating the timing of tumor inoculation and administration of treatments in BALB/c nude mice (*n* = 8 per group). **B** Tumor growth curves representing the progression of Huh7^SR^ cell-induced tumors in mice treated with sorafenib. **C**, **D** Representative images of tumors (**C**) harvested on day 27 post-treatment, and corresponding average tumor weights (**D**) in each group. **E** Expression levels of NCOA5, MYC and GPX4 in tumors. ns, not significant; ***P < 0.001 by one-way ANOVA with post hoc intergroup comparisons.

We subsequently investigated the in vivo effects of NCOA5 reduction on ferroptosis induction via the MYC–GPX4 signaling pathway. Huh7^SR^ cells transfected with different constructs were subcutaneously injected into BALB/c nude mice, followed by sorafenib treatment (10 mg/kg every three days for three weeks) (Fig. 6A). NCOA5 reduction significantly inhibited tumor growth, while the effect reversed by GPX4 overexpression (Fig. 6B-D). Notably, the levels ofNCOA5, GPX4, and MYC were significantly reduced in the xenograft tumor tissues from the shNCOA5 group compared to the control group and the GPX4-overexpressing group (Fig. 6E). These data collectively demonstrate the therapeutic potential of targeting NCOA5 to counteract sorafenib resistance in HCC via ferroptosis in vivo.

## Discussion

Hepatocellular carcinoma (HCC) remains a significant challenge in oncology due to its aggressive progression, complex biology, and high rate of therapy resistance [49, 50]. This study provides compelling evidence that Nuclear Receptor Coactivator 5 (NCOA5) plays a pivotal role in HCC progression and drug resistance. Importantly, our findings also reveal distinct roles for the SNCOA5 and non-SNCOA5 isoforms in HCC, further highlighting the complexity of NCOA5-mediated regulation. This study delineates a novel mechanism by which NCOA5, primarily through its shortened isoform SNCOA5, inhibits ferroptosis by upregulating glutathione peroxidase 4 (GPX4), thereby contributing to therapeutic resistance, particularly against sorafenib.

Our analyses confirmed that NCOA5 is significantly overexpressed in HCC tissues compared to adjacent normal tissues, with the SNCOA5 isoform driving the majority of this upregulation. In contrast, non-SNCOA5 expression was reduced in some HCC cases. These findings were validated through TCGA data and RT-qPCR analyses in clinical samples, which revealed that SNCOA5 mRNA levels were elevated in the majority of HCC tumors, while non-SNCOA5 levels were reduced in a subset of cases. This differential expression suggests that SNCOA5 and non-SNCOA5 play opposing roles in HCC. While SNCOA5 appears to promote tumor progression, as evidenced by its enrichment in tumor tissues and association with poor overall survival, non-SNCOA5 may act as a tumor suppressor, with its downregulation contributing to tumorigenesis.

The differential roles of these isoforms align with previous studies suggesting that full-length NCOA5 (non-SNCOA5) can act as a tumor suppressor in some contexts, inhibiting cancer cell proliferation and epithelial-to-mesenchymal transition (EMT) [25, 26, 29]. By contrast, SNCOA5, the truncated isoform, has been implicated in tumor promotion and progression [25, 26, 29]. Our data strongly support this dichotomy, as SNCOA5 expression was enriched in sorafenib-resistant HCC cell lines and patient samples, while non-SNCOA5 showed a decreasing trend. These findings emphasize the need to distinguish between SNCOA5 and non-SNCOA5 when evaluating the role of NCOA5 in HCC. Of note, the tumor-promoting role of SNCOA5 is likely a key driver of the oncogenic effects attributed to NCOA5 in HCC, making SNCOA5 a critical target for therapeutic intervention.

One of the key findings of this study is the role of NCOA5, particularly SNCOA5, in mediating resistance to sorafenib, the first FDA-approved tyrosine kinase inhibitor for advanced HCC [51]. High NCOA5 levels, driven by SNCOA5, were strongly associated with poor response to sorafenib in both clinical samples and sorafenib-resistant cell lines (Huh7^SR^ and BEL-7402^SR^). Mechanistically, we demonstrated that NCOA5 knockdown, which reduces both SNCOA5 and non-SNCOA5, sensitized resistant cells to sorafenib by promoting ferroptosis. This ferroptosis induction was mediated through the downregulation of GPX4, a key enzyme that protects cells from oxidative stress-induced ferroptosis. Gene Set Enrichment Analysis (GSEA) further revealed an inverse relationship between NCOA5 expression and ferroptosis signatures, and biochemical assays confirmed that NCOA5 knockdown increased reactive oxygen species (ROS) and lipid peroxidation while reducing glutathione (GSH) levels. These findings highlight the critical role of SNCOA5 in suppressing ferroptosis and promoting resistance to oxidative stress, thereby facilitating sorafenib resistance.

Previous studies have identified multiple pathways and factors contributing to sorafenib resistance in HCC. For instance, genetic mutations in genes such as BRAF, KDR (VEGFR2), and other components of the MAPK pathway can lead to altered sorafenib sensitivity [52]. And cancer cells can bypass the inhibitory effects of sorafenib by activating alternative signaling pathways, such as the PI3K/Akt/mTOR pathway, which can promote survival and proliferation despite treatment [53]. Moreover, the tumor microenvironment, including stromal cells, immune cells, and extracellular matrix, can contribute to resistance by secreting factors that promote cell survival and proliferation [54]. Our findings add a new dimension to the understanding of sorafenib resistance by highlighting the role of ferroptosis inhibition mediated by SNCOA5. SNCOA5-dependent ferroptosis complements existing knowledge and suggests that a multifaceted approach targeting several resistance mechanisms may be necessary for effective HCC treatment. Targeting SNCOA5, along with other pathways such as inhibiting GPX4 expression, could potentially overcome the limitations of current therapies and improve clinical outcomes.

Further investigation into the transcriptional regulation of GPX4 revealed that NCOA5 exerts its effects through the MYC signaling pathway. Our data showed that NCOA5 knockdown reduced MYC protein levels, which in turn downregulated GPX4 expression. Rescue experiments confirmed that MYC overexpression reversed the effects of NCOA5 knockdown on GPX4 and ferroptosis. Promoter-binding assays further demonstrated that MYC directly binds to the GPX4 promoter, establishing the NCOA5-MYC-GPX4 axis as a critical regulatory pathway in HCC. Notably, MYC is a well-characterized oncogene implicated in cell proliferation, metabolism, and tumor stemness [55, 56], and its regulation by NCOA5 further emphasizes the role of this axis in promoting HCC progression and therapy resistance.

In vivo studies substantiate the therapeutic potential of targeting NCOA5. Reduction of NCOA5 in sorafenib-resistant xenograft models significantly inhibited tumor growth and enhanced sorafenib efficacy by promoting ferroptosis. These findings provide strong preclinical evidence that targeting NCOA5, particularly SNCOA5, could overcome drug resistance and improve HCC treatment outcomes. Despite these promising results, this study has certain limitations. Larger, independent cohorts and prospective clinical trials are needed to validate the clinical utility of targeting NCOA5 in HCC. Additionally, while our study elucidates the NCOA5-MYC-GPX4 axis as a key regulatory pathway, the broader network of interactions and potential compensatory mechanisms within the tumor microenvironment remain to be fully explored. Importantly, future studies should aim to further investigate the distinct roles of SNCOA5 and non-SNCOA5, as these isoforms may have divergent implications for HCC pathogenesis and therapy.

In conclusion, this study reveals the multifaceted role of NCOA5 in HCC, particularly the tumor-promoting and drug-resistance roles of SNCOA5, and the potential tumor-suppressive role of non-SNCOA5. By elucidating the NCOA5-MYC-GPX4 axis, this study provides critical insights into the molecular mechanisms underlying HCC progression and resistance to sorafenib. Targeting SNCOA5 or modulating the NCOA5-MYC-GPX4 axis holds promise as a therapeutic strategy for overcoming drug resistance in HCC. As the treatment landscape for HCC continues to evolve, integrating personalized approaches based on the distinct molecular profiles of SNCOA5 and non-SNCOA5 may offer significant improvements in patient outcomes and survival rates.

## Materials and methods

### Immunohistochemical Analysis

Immunohistochemistry (IHC) staining was performed as described [57]. Briefly, 4 μm sections from the formalin-fixed paraffin-embedded tissues obtained from 78 HCC patients and their adjacent normal tissues were processed. All human tissue samples were collected under protocols approved by the Medical Ethics Committee of the First Afliated Hospital of Anhui Medical University (Approval No. 20040158), and written informed consent was obtained from all patients. The sections were stained with antibody against NCOA5 (Cat. No. 20175-1-AP, Proteintech; dilution 1:200). Sections incubated with rabbit IgG as the primary antibody served as negative controls, and a known IHC positive slide was utilized as a positive control. The percentage of positively stained cells was scored according to the following scale: 0, no staining observed in any field; 1, ≤10%; 2, 11–50%; 3, 51–75%; 4, >75%. Staining intensity was assessed using these criteria: 1 + , weak staining; 2 + , moderate staining; 3 + , strong staining. The percentage (P) and intensity (I) of nuclear, cytoplasmic, or membrane expression were multiplied to yield a numerical score (S = P • I). IHC images were acquired using the Leica DMi8 imaging system.

### Western blotting analysis

Total protein was extracted using a radioimmunoprecipitation assay (RIPA) lysis buffer supplemented with a protease inhibitor cocktail. Protein concentrations were quantified and normalized using a BCA assay kit (Thermo Fisher Scientific, USA). Proteins from each group were loaded onto sodium dodecyl sulfate-polyacrylamide gel electrophoresis (SDS-PAGE) gels for separation, followed by transfer to polyvinylidene fluoride (PVDF) membranes (Millipore). The membranes were incubated with primary antibody against NCOA5 (Cat. No. 20175-1-AP, Proteintech; dilution 1:1000), SLC7A11 (cat. No. 26864-1-AP, Proteintech; dilution 1:1000), GPX4 (cat. No. 67763-1-Ig, Proteintech; dilution 1:1000), MYC (cat. No. 10828-1-AP, Proteintech; dilution 1:1000), E2F1 (cat. No. 66515-1-Ig, Proteintech; dilution 1:1000), and β-actin (catalog no. 4970, Cell Signaling Technology; dilution 1:2000), at 4 °C overnight. β-actin was chosen as the housekeeping gene due to its stable expression across the experimental conditions, which was validated by prior pilot studies. Subsequently, the membranes were incubated with the secondary antibody at 25 °C for 1 h. Immunoblots were developed using a chemiluminescent reagent (Beyotime, China) according to the manufacturer’s instructions. The intensity of each protein band was normalized to β-actin as a loading control.

### Reverse Transcription-quantitative PCR (RT-qPCR)

Total RNA was extracted from cells using TRIzol (Thermo Fisher). Two micrograms of RNA were reverse transcribed with the High-Capacity cDNA Reverse Transcription Kit (Applied Biosystems, USA) following the manufacturer’s instructions. An equal quantity of cDNA was then amplified and quantified using the SYBR Green PCR amplification kit (Thermo Fisher) in the Applied Biosystems 7500 system. The cDNA was analyzed through both semi-quantitative PCR and quantitative real-time PCR (RT-qPCR) using the following primers: S*NCOA5* forward: 5’- TCTCTGCCTGGTGAGCTACGT -3’ and reverse: 5’-CTGGCTGTTTGCTGCTGTGGA-3’; non-SNCOA5 forward: 5’-TCTCTGCCTGGCCCGATTTCCCG-3’ and reverse: 5’-CTGGCTGTTTGCTGCTGTGGA-3’; *β-actin* forward: 5’-GACTTATGATGGATCACAGGTTGA-3’ and reverse: 5’-ATAAAAGAGAGGCAGGCGCT-3’.

### Cells and cell culture

Huh7 and BEL-7402 cells were obtained from the American Type Culture Collection (ATCC). These cells were cultured in DMEM medium supplemented with 10% FBS at 37 °C in a humidified atmosphere with 5% CO_2._ The sorafenib-resistant sublines Huh7^SR^ and BEL-7402^SR^ were established and cultured under the continuous presence sorafenib. All cell lines have been authenticated using short tandem repeat DNA profiling (Beijing Microread Genetics Co., Ltd., Beijing, China). All cell lines underwent routine PCR testing for mycoplasma infections.

### Establishment of sorafenib resistance

Initially, we ascertained the IC50 values for Huh7 and BEL-7402 cells in response to sorafenib. The cells were placed in a 96-well plate and exposed to escalating concentrations of the drug. Following a three-day period, the cells underwent CCK8 assay to assess their viability, as detailed later. Subsequently, Huh7 and BEL-7402 cells were cultured in 6-well plates, with each well containing 50,000 cells, and were treated with sorafenib concentrations slightly lower than their respective IC50 values. Over the ensuing weeks, the sorafenib concentration was gradually augmented by increments of 0.25 μM each time. Through this process, spanning several months, we successfully generated two sorafenib-resistant cell lines from Huh7 (designated as Huh7^SR^) and BEL-7402 (designated as BEL-7402^SR^). Once these resistant cell lines were established, they were maintained in continuous culture with the presence of sorafenib.

### Generation of Stable Cell Lines

The full-length cDNA of NCOA5 was cloned into the pCDNA3.1 flag plasmid. The shRNA targeting human NCOA5 (target sequence #1: GCGTAGAGAAGAGCTTTATCG; target sequence #2: GGAGACAGTCGAGATTCAAGG) was cloned into pLKO.1-U6-EGFP-puro plasmid. Transfection of plasmids or LNA GapmeR was performed using Lipofectamine 3000 (Thermo Fisher Scientific) according to the manufacturer’s instructions.

### Malondialdehyde assay

The cells were collected, homogenized, and lysed. Protein concentrations in each sample were measured using the Pierce™ BCA protein assay kit (23225, Thermo Fisher Scientific, USA). Subsequently, lipid peroxidation levels were assessed using the MDA assay kit (S0131S; Beyotime Biotechnology, China) following the manufacturer’s guidelines.

### Glutathione assay

The total glutathione (GSH) levels were quantified using a GSH Assay Kit (#BC1175, Solarbio) and normalized based on cell count, following the manufacturer’s instructions.

### ROS levels

The experiments were carried out according to a standardized protocol. Briefly, cells were harvested, and 1 mL of diluted DCFH (Beyotime, S0033S-1) was added to the cell suspension. This mixture was incubated at 37 °C for 21 minutes, after which the cells were washed three times with serum-free medium. The cell pellet was then resuspended in 500 μL of PBS and treated with the ROS-positive control (Beyotime, S0033S-2) for 20 minutes. The resulting mixture was subsequently analyzed using a flow cytometer.

### Cell viability assay

The cell lines were dispersed into 96-well plates at a concentration of 2000 cells/100 uL and subjected to sorafenib treatment at concentrations of 1, 10, 20, 40, 80, 100 ug/mL for a period of 48 hours. Subsequently, CCK-8 solution (provided by MedChem Express) was introduced into each well at the conclusion of the experiment. Following a 3-hour incubation period, the optical density (OD) values were evaluated using a Bio-Tek microplate reader at a wavelength of 450 nm.

### Gene set enrichment analysis

Microarray data (accession no. GSE25097, GSE109211) were obtained from the Gene Expression Omnibus of NCBI (https://www.ncbi.nlm.nih.gov/geo/) and subjected to Gene set enrichment analysis (GSEA) using GSEA software (version 2.0.13; https://www.broadinstitue.org/gsea/index. jsp).

### Animal experiments

All animal experiments were approved by the Animal Care and Use Committee (IACUC) of Bengbu Medical University (Approval No. 2024383) and conducted in accordance with ethical guidelines. Five-week-old female nude BALB/c nude mice were purchased from Vital River Laboratory Animal Technology. Huh7^SR^-shCtrl or Huh7^SR^-shNCOA5 (5 × 10^6^ cells) mixed with PBS (100 ul/each mice) was subcutaneously injected into the flanks of mice. When tumor volumes reached approximately 100 mm^3^, mice were treated with sorafenib (10 mg/kg, formulated in 0.5% carboxymethylcellulose solution, administered every 3 days) based on established protocols [58]. The tumor volume was measured every 3 days, and the volume was estimated according to the following formula: volume = (length × width^2^)/2. The mice were sacrificed at the end of the experiment, and the xenografts were resected, weighed, and photographed. The sorafenib dosage and administration frequency were selected based on its established efficacy and tolerability in similar mouse models reported in the literature [58–60].

### Statistical analysis

All statistical analyses were conducted utilizing SPSS version 17.0 (SPSS, Inc., Chicago, IL, USA). For pairwise comparisons, the Student’s t-test (or paired t-test) was employed, while for comparisons involving more than two groups, a one-way ANOVA was utilized, supplemented by posthoc analyses to discern intergroup differences. A *P*-value below 0.05 was deemed indicative of statistical significance.

## Supplementary information

supplementary table 1

original data figure 4 WB

original data figure 2 WB

original data figure 5 WB

## Data Availability

The data used to support the findings of this study are available upon reasonable request from the corresponding author.
