# Supplementary material for: NCOA5 induces sorafenib resistance in hepatocellular carcinoma by inhibiting ferroptosis
Source: Cell Death Discov. 2025 May 2;11:215. doi: 10.1038/s41420-025-02473-1 (PMC12052255; doi:10.1038/s41420-025-02473-1)
Supplement: Supplementary file 1 — supplementary table 1 [file 41420_2025_2473_MOESM1_ESM.docx]

| Variables |  | NCOA5 level | | | *P* |
| --- | --- | --- | --- | --- | --- |
|  | *n* | Low | High |  | |
| Patients | 78 | 31 | 47 |  | |
| Gender |  |  |  |  | |
| Male | 42 | 22 | 20 | 0.345 | |
| Female | 36 | 15 | 21 |  | |
| Age (years) | 78 | 55.32±1.60 | 58.61±1.43 | 0.761 | |
| ALT |  |  |  |  | |
| ≤40 U/L | 27 | 14 | 13 | 0.687 | |
| ＞40 U/L | 51 | 24 | 27 |  | |
| AFP |  |  |  |  | |
| ≤400 ng/ml | 24 | 11 | 13 | 0.502 | |
| ＞400 ng/ml | 53 | 20 | 33 |  | |
| Cirrhosis |  |  |  |  | |
| No | 30 | 18 | 12 | 0.115 | |
| Yes | 48 | 20 | 28 |  | |
| Tumor size |  |  |  |  | |
| ≤5 cm | 21 | 9 | 12 | 0.0346 | |
| ＞5 cm | 57 | 11 | 46 |  | |
| Stage |  |  |  |  | |
| I-II | 37 | 21 | 16 | 0.358 | |
| III | 41 | 19 | 22 |  | |

**Supplementary Table 1** Analysis of the correlations between NCOA5 with the clinicopathological parameters of HCC.

For categorical variables, the number of patients was shown as *n*; quantitive variables were mean ± SD.
