# Supplementary figures and images for: NCOA5 induces sorafenib resistance in hepatocellular carcinoma by inhibiting ferroptosis
Source: Cell Death Discov. 2025 May 2;11:215. doi: 10.1038/s41420-025-02473-1 (PMC12052255; doi:10.1038/s41420-025-02473-1)

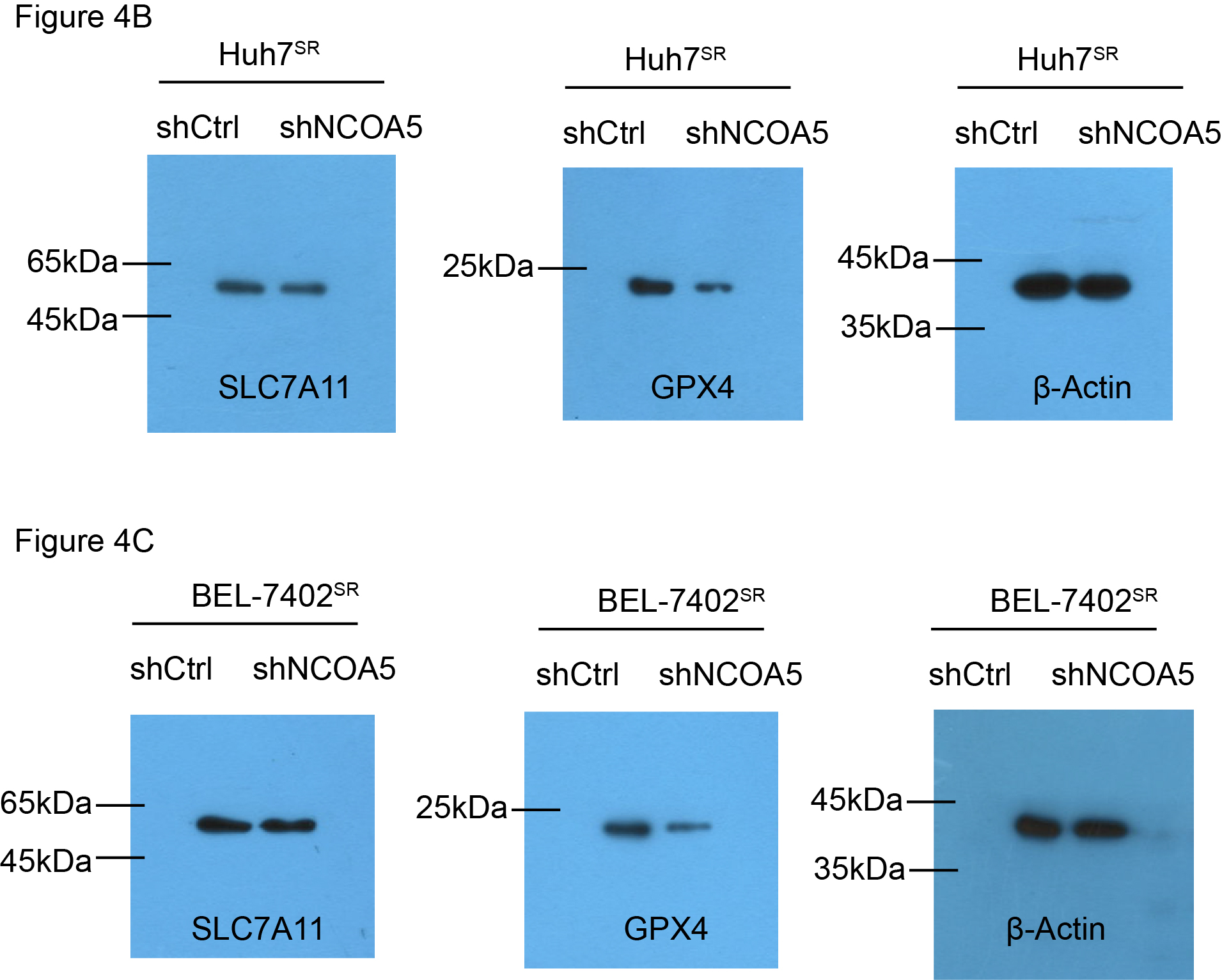

Supplement: Supplementary file 2 — original data figure 4 WB [file 41420_2025_2473_MOESM2_ESM.jpg]

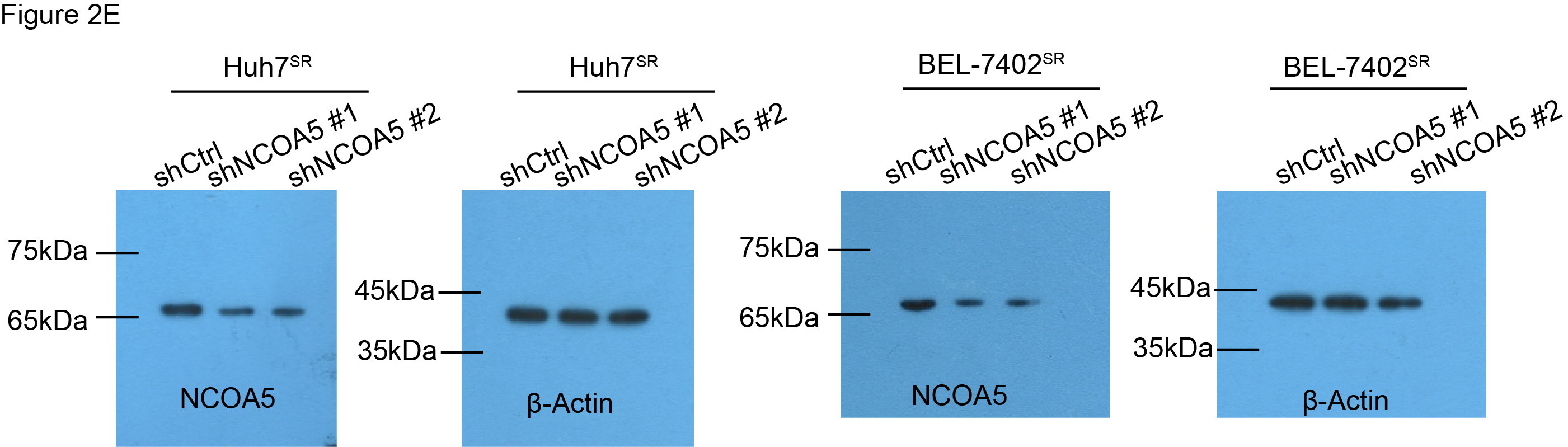

Supplement: Supplementary file 3 — original data figure 2 WB [file 41420_2025_2473_MOESM3_ESM.jpg]

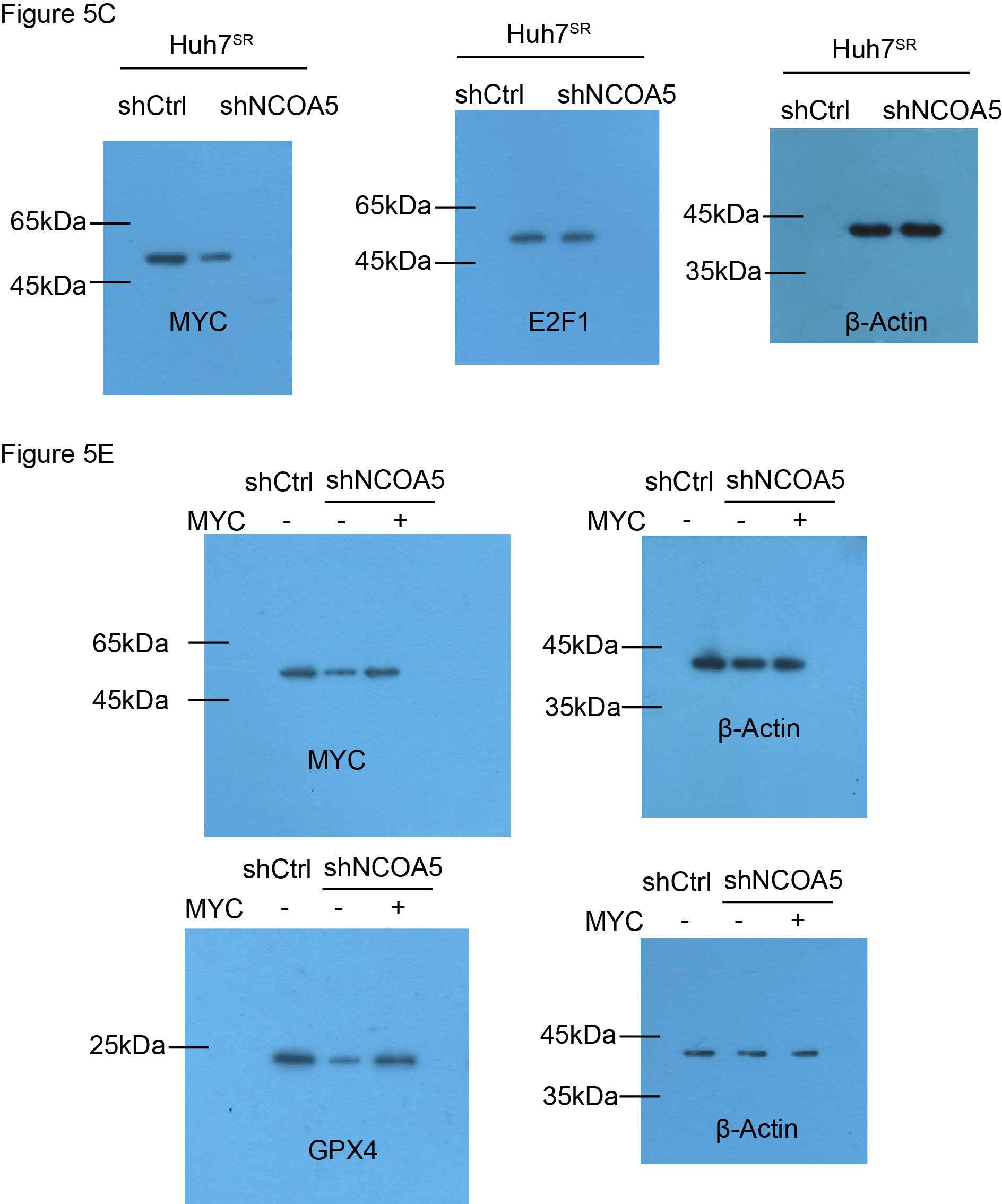

Supplement: Supplementary file 4 — original data figure 5 WB [file 41420_2025_2473_MOESM4_ESM.jpg]
